# Clinical and economic burdens experienced by patients with painful diabetic peripheral neuropathy: An observational study using a Japanese claims database

**DOI:** 10.1371/journal.pone.0187250

**Published:** 2017-10-27

**Authors:** Nozomi Ebata-Kogure, Kazutaka Nozawa, Aya Murakami, Tetsumi Toyoda, Yuri Haga, Koichi Fujii

**Affiliations:** 1 Pfizer Japan, Tokyo, Japan; 2 Department of Public Health, Aichi Medical University School of Medicine, Nagakute, Japan; 3 Clinical Study Support, Inc., Nagoya, Japan; Weill Cornell Medical College in Qatar, QATAR

## Abstract

**Background:**

Diabetic peripheral neuropathy (DPN) may often be painful. Despite the high prevalence of painful DPN (pDPN) among patients with diabetes mellitus (DM), understanding of its clinical and economic burden is limited. This study aimed to describe the clinical and economic burdens faced by patients with pDPN in Japan, and compared them with those experienced by patients with DPN but without painful symptoms (non-pDPN).

**Methods:**

This retrospective, observational study used data from a large-scale, hospital-based Japanese claims database collected from April 2008 to June 2015. Comorbidities, clinical departments visited, length of hospital stay, and medical costs for the period of ± 6 months from the diagnosis of pDPN or non-pDPN were described for each group. Glycemic control status was examined for each group for patients with glycated hemoglobin data.

**Results:**

The data of 8,740 patients with pDPN (mean age 70.0 years, 53.4% male) and 12,592 patients with non-pDPN (mean age 67.7 years, 55.7% male) were analyzed. Patients with pDPN had more comorbidities than patients with non-pDPN; 48.7% and 30.9% of patients in the respective groups had 20 or more comorbidities. The median length of hospital stay was 5 days longer in patients with pDPN. The median total medical costs were higher in patients with pDPN (\517,762) than in patients with non-pDPN (\359,909). Patients with pDPN spent higher median costs for medications, but the costs for glycemic control drugs were similar in both groups. For 3,372 patients with glycated hemoglobin data, glycemic control was similar between the two groups.

**Conclusion:**

Patients with pDPN experienced greater clinical and economic burdens than patients with non-pDPN, suggesting that patients who develop pDPN may suffer not only from the complications of DM and pain, but also from other comorbid disorders.

## Introduction

Diabetic peripheral neuropathy (DPN) is one of the most common complications of diabetes mellitus (DM). The prevalence of DPN was 22.4–28.5% in patients with DM in the north-west of England and the United States [1,2], and 27.6–36.7% in Japan [3,4]. The prevalence of DPN varied across the studies mainly because of differences in the study designs; however, these studies showed that many patients with DM suffer from DPN, which impairs patients’ quality of life and increases the risks for foot ulcers and lower extremity amputations [3,5,6].

Diabetic peripheral neuropathy is often accompanied by burning pain, electrical shock-like pain, lancinating pain, paresthesia or allodynia [7], which is known as painful DPN (pDPN). Patients with DM often report foot pain, even if the formal diagnosis of DPN has not been made: an online survey of 1,004 patients with DM in the US found that 83% of respondents reported symptoms of pDPN, but only 41% had been diagnosed with DPN [8]. A Japanese study of 298 patients with DM reported that 22.1% had pDPN, but only 36.4% of these patients’ physicians had recognized their pDPN [9]. The same study also showed that pDPN impaired patients’ quality of life and mental state. These studies suggest that pDPN may be under-diagnosed and under-treated in clinical settings, despite its high prevalence and negative impacts on patients.

The under-diagnosis of pDPN may reflect that physicians infrequently undertake detailed clinical examinations, possibly because of limited knowledge of the disease burden. Outside Japan, several studies of claims databases have separately reported that DPN and pDPN are associated with comorbid conditions and high medical costs [10–13], but a direct comparison of patients with DPN and patients with pDPN has not been made. In Japan, one study has examined the influences of DPN on medical costs using a claims database [14], but the clinical and economic burdens of pDPN have not been reported in a Japanese population.

The aims of this study were therefore to illuminate the clinical and economic burdens of pDPN using a large-scale claims database in Japan, and to compare the characteristics of patients with pDPN and patients with DPN without painful symptoms (non-pDPN).

## Methods

### Data source

This retrospective, observational database study used a hospital-based claims database constructed by Medical Data Vision Co., Ltd. (MDV, Tokyo, Japan), which has been available since April 2008. The database included the data of approximately 14 million patients admitted to 238 hospitals across Japan. As of February 2016, these hospitals covered about 15% of all Japanese acute-care hospitals using the Diagnosis Procedure Combination/Per-Diem Payment System (DPC/PDPS). The database contains anonymized data including age, sex, diagnoses according to the 10th revision of the International Classification of Diseases and Related Health Problems (ICD-10) codes and Japanese Disease Name Codes [15], procedures, prescriptions and inpatient/outpatient status. Laboratory data were available from a limited number of hospitals. The database has a similar distribution of age and sex to that of national patient statistics in Japan [16], and it has been used for various epidemiological studies [17–19]. This study used all data available, which were data collected from April 1, 2008 (when MDV first started to offer this claims database) to June 30, 2015.

### Disease definition

In this study, DPN without painful symptoms is referred to as non-pDPN. Thus, DPN was defined as either pDPN or non-pDPN. Patients with pDPN were defined as patients with type 2 DM who had a record of any of the following diseases: peripheral neuropathic pain (Japanese Disease Name Code: 8846220), neuropathic pain (8847489), or diabetic neuropathic pain (8848768). Patients with non-pDPN were defined as patients with type 2 DM who had a record of any of the following diseases: peripheral neuropathy (8840255), diabetic peripheral neuropathy (2505018) or type 2 diabetic peripheral neuropathy (8845100).

### Patients

Patients with a definite diagnosis of type 2 DM (ICD-10 codes E11‒14) between April 1, 2008 and June 30, 2015 were identified from the database. Of these, patients with a first diagnosis record of pDPN or non-pDPN at age ≥18 years were extracted. Patients with type 1 DM (ICD-10 code E10), cancer (identified with the diagnostic terms of ICD-10 containing “*gan* [carcinoma]”, “*shinseibutsu* [neoplasm]” or “*shuyô* [tumor]”) or cancer pain (Japanese Disease Name Code: 7998003) were excluded. Patients in whom the first diagnosis of pDPN or non-pDPN was made before the diagnosis of type 2 DM, patients lost to follow-up after the first diagnosis of pDPN or non-pDPN, and patients lacking data for ≥6 months before or after the first diagnosis of pDPN or non-pDPN were also excluded.

### Measures

For patient characteristics, the following data in the month of the first diagnosis of pDPN or non-pDPN were collected: age, sex, duration of DM, comorbidities, and medications. In addition, glycated hemoglobin (HbA1c) concentration data were collected for patients in whom HbA1c was measured within 30 days before or after the first diagnosis of pDPN or non-pDPN. The duration of DM was measured from the earliest date of diagnosis of DM in a patient’s record to the date of the first diagnosis of pDPN or non-pDPN.

For clinical burden, the following data for the period of ± 6 months from the first diagnosis of pDPN or non-pDPN were examined for each group: number of comorbidities, comorbid diagnoses, number and type of clinical departments visited, and the length of hospital stay.

For economic burden, the following data for the period of ± 6 months from the first diagnosis of pDPN or non-pDPN were examined for each group: total medical costs, costs of each medical service provided (including drugs), cost breakdown by clinical department and separate breakdown of costs by type of drug. The categories of medical services included outpatient, medications, injections, procedures, examinations, hospitalization, surgery, anesthesia, home medical care and others. In this study, “total medical costs” were the all-cause medical costs that patients paid to the hospitals, regardless of whether they were incurred by pDPN or non-pDPN.

Drugs were categorized as follows: antidiabetic drugs (glycemic control drugs and any other antidiabetic drugs), analgesics (non-steroidal anti-inflammatory drugs [NSAIDs]/acetaminophen, drugs for neuropathic pain and any other analgesics), anesthesia and any other drugs. Glycemic control drugs included insulin, glucagon-like peptide-1 (GLP-1) receptor agonists, sulfonylurea (SU) agents, biguanides, insulin sensitizers, alpha glucosidase inhibitors, rapid-acting insulinotropic agents, dipeptidyl peptidase-4 (DPP-4) inhibitors, sodium-glucose co-transporter-2 (SGLT2) inhibitors and compounding agents (S1 Table).

### Ethics statement

This was an observational study using de-identified claims data provided by MDV (a commercial database provider), and data did not contain any information allowing identification of a patient. The Japanese Ethical Guidelines for Medical and Health Research Involving Human Subjects do not apply to a study exclusively using de-identified data, thus the study was exempt from the requirement for acquisition of informed consent.

### Statistical analysis

Patient characteristics were summarized descriptively for the pDPN and non-pDPN groups. For patients with HbA1c data, patient characteristics including the HbA1c concentration and the use of antidiabetic drugs were additionally summarized separately from the overall study population. For the clinical and economic burden, basic statistics were calculated for each measure in each group. Continuous variables were expressed as the mean with standard deviation (SD) or the median with interquartile range. Categorical variables were expressed as the number and proportion (%). The two groups were compared using the t-test for continuous variables and the chi-square test for categorical variables. All statistical analyses were performed using SAS release 9.4 (SAS Institute Inc., Cary, NC, USA).

## Results

### Patient characteristics

**Fig 1:**
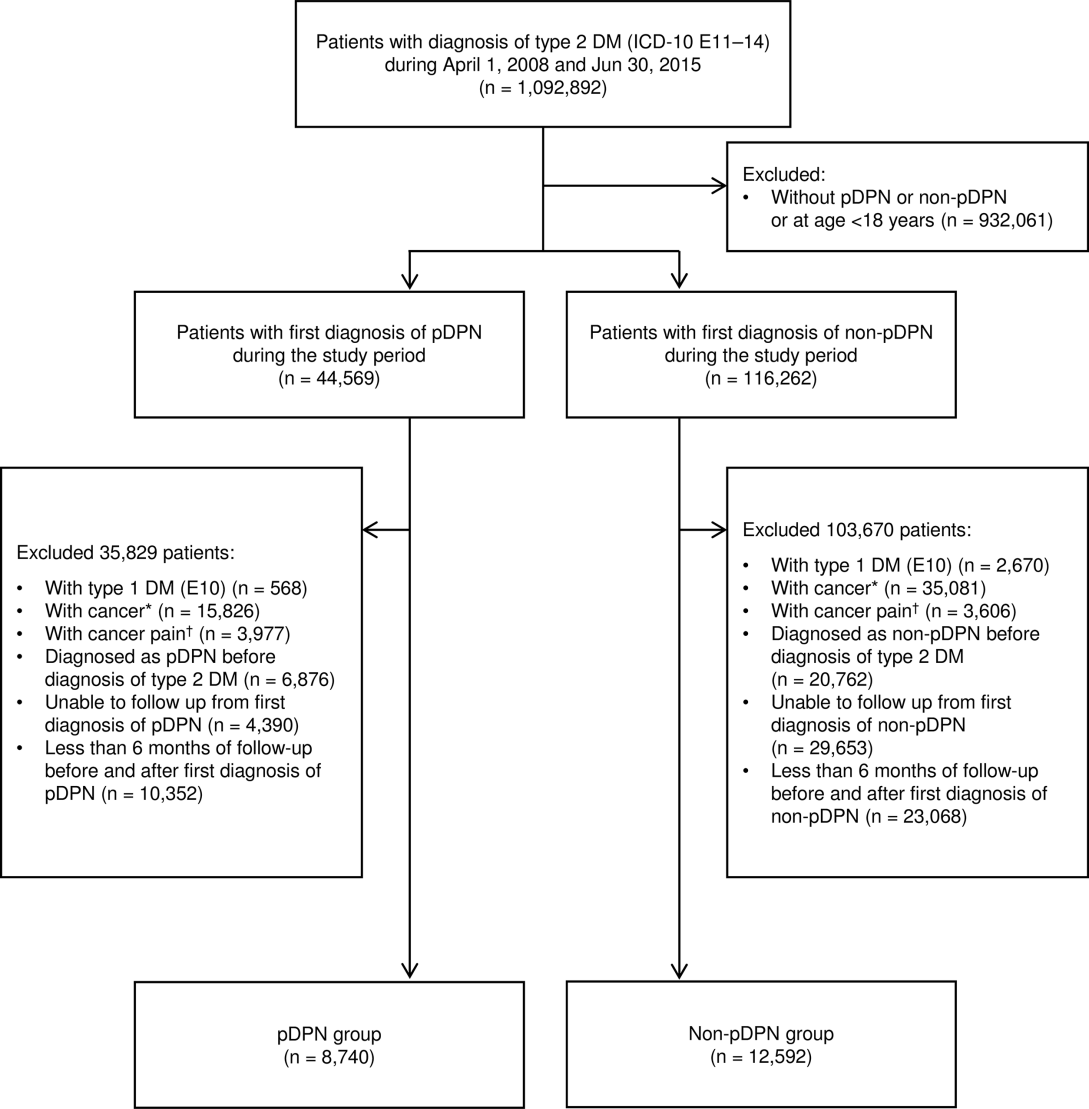
Flow chart showing the selection of the pDPN and non-pDPN study groups. Abbreviations: pDPN, painful diabetic peripheral neuropathy; non-pDPN, diabetic peripheral neuropathy without painful symptoms; DM, diabetes mellitus. *Identified by the ICD-10 diagnostic term containing “*gan* [carcinoma]”, “*shinseibutsu* [neoplasm]” or “*shuyô* [tumor]”. ^†^Identified by the Japanese Disease Name Code 7998003.

We identified 8,740 patients with pDPN and 12,592 patients with non-pDPN (Fig 1); their characteristics were summarized in Table 1. The pDPN group comprised a greater proportion of older patients than the non-pDPN group: the proportions of patients aged ≥70 years were 57.0% and 47.8%, respectively. The sex distributions were similar between the groups. The mean duration of DM was approximately 5 years in both groups. Patients with pDPN had more comorbidities than patients with non-pDPN. Lumbar spinal stenosis was more common in the pDPN group (33.4%) than the non-pDPN group (8.6%). Fewer patients with pDPN received antidiabetic drugs, but more patients received analgesics and anesthesia, compared with patients with non-pDPN.

**Table 1:** Patient characteristics for pDPN group and non-pDPN group (n = 21,332).

	Patients with pDPN		Patients with non-pDPN		p-value
	(n = 8,740)		(n = 12,592)		
Age (years)	70.0	(11.2)	67.7	(11.9)	<0.001
Age group, n (%)					
20–49	474	(5.4)	987	(7.8)	<0.001
50–59	963	(11.0)	1,770	(14.1)	
60–69	2,324	(26.6)	3,817	(30.3)	
70–79	3,263	(37.3)	4,044	(32.1)	
80–89	1,584	(18.1)	1,814	(14.4)	
90≤	132	(1.5)	160	(1.3)	
Male, n (%)	4,664	(53.4)	7,012	(55.7)	<0.001
Duration of DM (years)	5.1	(5.3)	5.0	(5.4)	<0.001
Number of comorbidities, n (%)					
1–4	870	(10.0)	1,994	(15.9)	<0.001
5–9	2,226	(25.6)	4,334	(34.5)	
10–19	3,729	(42.9)	4,849	(38.6)	
20≤	1,877	(21.6)	1,375	(11.0)	
Top 10 most common comorbidities*, n (%)					
Hypertension	4,622	(52.9)	6,932	(55.1)	0.002
Hyperlipidemia	2,298	(26.3)	3,474	(27.6)	0.036
Constipation	2,761	(31.6)	2,930	(23.3)	<0.001
Insomnia	2,507	(28.7)	2,575	(20.4)	<0.001
Low back pain	2,406	(27.5)	2,127	(16.9)	<0.001
Gastric ulcer	1,980	(22.7)	2,314	(18.4)	<0.001
Lumbar spinal stenosis	3,005	(34.4)	1,082	(8.6)	<0.001
Hypercholesterolemia	1,454	(16.6)	2,340	(18.6)	<0.001
Chronic gastritis	1,867	(21.4)	1,878	(14.9)	<0.001
Angina	1,564	(17.9)	2,083	(16.5)	0.010
Use of medications^†^, n (%)					
Yes	8,669	(99.6)	12,229	(97.4)	<0.001
Antidiabetic drugs					
*Glycemic control drugs*	2,965	(34.1)	5,968	(47.5)	<0.001
*Any other antidiabetic drugs*	2,115	(24.3)	8,111	(64.6)	<0.001
Analgesics					
*NSAIDs/acetaminophen*	5,361	(61.6)	4,033	(32.1)	<0.001
*Drugs for neuropathic pain*	8,304	(95.4)	1,112	(8.9)	<0.001
*Any other analgesics*	818	(9.4)	701	(5.6)	<0.001
Anesthesia	1,649	(18.9)	1,449	(11.5)	<0.001
Any other drugs	7,827	(89.9)	11,114	(88.5)	0.001

For each measure, data recorded in the month of the first diagnosis of pDPN or non-pDPN were summarized. Data were expressed as mean (SD) or number (%). P-values were calculated using the t-test for continuous variables and the chi-square test for categorical variables.

Abbreviations: pDPN, painful diabetic peripheral neuropathy; non-pDPN, diabetic peripheral neuropathy without painful symptoms; DM, diabetes mellitus; NSAIDs, nonsteroidal anti-inflammatory drugs.

*The 10 most common comorbidities in the study population were listed.

^†^Data were not available for 74 patients (37 patients for each group); the percentages were calculated using the denominators of 8,703 for the pDPN group and 12,555 for the non-pDPN group.

Treatment status was further analyzed for 3,372 patients (939 patients with pDPN and 2,433 patients with non-pDPN) who had HbA1c data (Table 2). The age and sex distributions of the 939 patients with pDPN were similar to those of the overall pDPN cohort. The 2,433 patients with non-pDPN and HbA1c data included fewer older patients (the proportion of patients aged ≥70 years was 36.2%) and more men than the overall non-pDPN cohort. In both groups, the mean duration of DM was 1.7 years longer than that of the corresponding overall patient cohort. The glycemic control status was similar between the groups: the mean (SD) HbA1c concentrations were 6.9 (1.3)% in the pDPN group and 7.0 (1.2)% in the non-pDPN group. Nearly 40% of patients in both groups had a HbA1c concentration ≥7.0%, which was interpreted as poor glycemic control. More patients with non-pDPN (75.6%) received glycemic control drugs (of any kind) than patients with pDPN (55.9%).

**Table 2:** The use of antidiabetic drugs in patients with HbA1c data (n = 3,372).

	Patients with pDPN		Patients with non-pDPN		p-value
	(n = 939)		(n = 2,433)		
Age (years)	69.3	(10.9)	65.0	(11.7)	<0.001
Male, n (%)	508	(54.1)	1,484	(61.0)	<0.001
Duration of DM (years)	6.8	(6.3)	6.7	(6.4)	0.624
HbA1c levels ≥ 7%*, n (%)	354	(37.7)	972	(40.0)	0.230
The use of antidiabetic drugs^†^, n (%)					
Glycemic control drugs	523	(55.9)	1,835	(75.6)	<0.001
*Insulin*	220	(23.5)	537	(22.1)	0.388
*GLP-1 receptor agonists*	12	(1.3)	18	(0.7)	0.135
*SU agents*	132	(14.1)	402	(16.6)	0.081
*Biguanides*	161	(17.2)	968	(39.9)	<0.001
*Insulin sensitizers*	49	(5.2)	172	(7.1)	0.052
*Alpha glucosidase inhibitors*	110	(11.8)	310	(12.8)	0.425
*Rapid-acting insulinotropic agents*	28	(3.0)	157	(6.5)	<0.001
*DPP-4 inhibitors*	247	(26.4)	822	(33.9)	<0.001
*SGLT2 inhibitors*	4	(0.4)	4	(0.2)	0.161
*Compounding agents*	17	(1.8)	12	(0.5)	<0.001
Any other antidiabetic drugs	225	(24.0)	735	(30.3)	<0.001

For each measure, data recorded in the month of the first diagnosis of pDPN or non-pDPN were summarized. Data were expressed as mean (SD) or number (%). P-values were calculated using the t-test for continuous variables and the chi-square test for categorical variables.

Abbreviations: pDPN, painful diabetic peripheral neuropathy; non-pDPN, diabetic peripheral neuropathy without painful symptoms; DM, diabetes mellitus; GLP-1, glucagon-like peptide-1; SU, sulfonylurea; DPP-4, dipeptidyl peptidase-4; SGLT2, sodium-glucose co-transporter-2.

*An HbA1c concentration ≥7% was considered to represent poor glycemic control.

^†^Data were not available for three patients in the pDPN group and five patients in the non-pDPN group; the percentages were calculated using the denominators of 936 for the pDPN group and 2,428 for the non-pDPN group.

### Clinical burden

Table 3 summarized the clinical burdens experienced by each group. Patients with pDPN had more comorbidities than patients with non-pDPN: the proportions of patients with ≥20 comorbidities were 48.7% and 30.9%, respectively. Among the 10 most commonly observed comorbidities in this study, the proportions of patients who had lumbar spinal stenosis, low back pain, insomnia, constipation or chronic gastritis were >10% higher in patients with pDPN than in patients with non-pDPN. For the clinical departments visited, 70.9% of patients with pDPN visited the department of orthopedic surgery. More patients with non-pDPN (17.5%) visited the department of otorhinolaryngology than patients with pDPN (10.6%). The median length of hospital stay was 5 days longer in patients with pDPN than in patients with non-pDPN.

**Table 3:** Clinical burdens experienced by patients with pDPN and patients with non-pDPN (n = 21,332).

	Patients with pDPN		Patients with non-pDPN		p-value
	(n = 8,740)		(n = 12,592)		
Number of comorbidities, n (%)					
1–4	130	(1.5)	542	(4.3)	<0.001
5–9	949	(10.9)	2,557	(20.3)	
10–19	3,408	(39.0)	5,601	(44.5)	
20≤	4,253	(48.7)	3,892	(30.9)	
Top 10 most common comorbidities*, n (%)					
Hypertension	5,726	(65.5)	8,123	(64.5)	0.130
Constipation	3,756	(43.0)	4,055	(32.2)	<0.001
Hyperlipidemia	2,970	(34.0)	4,320	(34.3)	0.622
Insomnia	3,305	(37.8)	3,345	(26.6)	<0.001
Low back pain	3,128	(35.8)	2,946	(23.4)	<0.001
Gastric ulcer	2,719	(31.1)	3,141	(24.9)	<0.001
Chronic gastritis	2,463	(28.2)	2,594	(20.6)	<0.001
Hypercholesterolemia	1,963	(22.5)	2,962	(23.5)	0.070
Angina	2,130	(24.4)	2,742	(21.8)	<0.001
Lumbar spinal stenosis	3,399	(38.9)	1,414	(11.2)	<0.001
Number of clinical department visited, n (%)					
1	1,174	(13.4)	2,369	(18.8)	<0.001
2	2,296	(26.3)	3,892	(30.9)	
3	2,185	(25.0)	2,999	(23.8)	
4	1,437	(16.4)	1,759	(14.0)	
5≤	1,648	(18.9)	1,573	(12.5)	
Commonly visited departments^†^, n (%)					
Internal medicine	4,860	(55.6)	7,870	(62.5)	<0.001
Orthopedic surgery	6,195	(70.9)	4,266	(33.9)	<0.001
Ophthalmology	2,591	(29.6)	4,211	(33.4)	<0.001
Dermatology	1,852	(21.2)	2,057	(16.3)	<0.001
Otorhinolaryngology	928	(10.6)	2,198	(17.5)	<0.001
Cardiology	1,100	(12.6)	1,652	(13.1)	0.253
Neurosurgery	1,035	(11.8)	1,332	(10.6)	0.004
Urology	1,108	(12.7)	1,240	(9.8)	<0.001
Neurology	996	(11.4)	1,136	(9.0)	<0.001
Diabetology	360	(4.1)	1,140	(9.1)	<0.001
Length of hospital stay^‡^ (days)					
Mean (SD)	37.1	(44.5)	29.8	(37.1)	<0.001
Median (25th–75th percentiles)	22	(10–46)	17	(9–36)	

For each measure, data for the period of ± 6 months from the first diagnosis of pDPN or non-pDPN were summarized. P-values were calculated using the t-test for continuous variables and the chi-square test for categorical variables.

Abbreviations: pDPN, painful diabetic peripheral neuropathy; non-pDPN, diabetic peripheral neuropathy without painful symptoms; SD, standard deviation.

*The 10 most common comorbidities in the study population were listed.

^†^Clinical departments visited by >1,000 patients in either group were listed.

^‡^Length of hospital stay was calculated as a sum of the number of days of hospitalization during the period of ± 6 months from the first diagnosis of pDPN or non-pDPN.

### Economic burden

Table 4 summarized the medical costs per patient for each group. The median total medical costs were higher in patients with pDPN (\517,762) than in patients with non-pDPN (\359,909). The first and second highest median costs by service were those for hospitalization and surgery in both groups: a slightly higher proportion of pDPN group incurred costs for these services compared with the non-pDPN group (hospitalization 39.8% *versus* 34.1%, surgery 24.9% *versus* 18.9%, respectively). The first and second largest differences in the median costs by service between the groups were observed for hospitalization and medications, and the median costs for these two services were \112,700 and \66,187 higher in patients with pDPN than in patients with non-pDPN, respectively. Although the median costs for medications were higher in the pDPN group, the breakdown of costs by drug type showed that the median costs for glycemic control drugs were similar (approximately \56,000 in each group).

**Table 4:** Medical costs per patient for the pDPN and non-pDPN groups (n = 21,332).

	pDPN group (n = 8,740)					Non-pDPN group (n = 12,592)				
			Costs (\)					Costs (\)		
	n	(%)	25th percentile	Median	75th percentile	n	(%)	25th percentile	Median	75th percentile
**Total medical costs**	8,740	(100.0)	265,039	517,762	1,286,214	12,592	(100.0)	181,646	359,909	842,144
**Costs by resource used**										
***A*. *Medical services***										
Outpatient	8,707	(99.6)	7,700	11,890	18,120	12,548	(99.7)	5,600	9,050	13,820
Medications	8,732	(99.9)	96,523	196,969	325,218	12,417	(98.6)	55,811	130,782	233,823
Injections	4,623	(52.9)	2,721	12,550	51,239	5,383	(42.7)	1,966	9,547	40,738
Procedures	4,093	(46.8)	1,438	4,082	12,737	4,344	(34.5)	928	2,928	12,952
Examinations	8,637	(98.8)	54,220	97,022	168,112	12,512	(99.4)	49,340	80,275	141,653
Hospitalization	3,479	(39.8)	263,960	532,300	1,001,128	4,300	(34.1)	227,958	419,600	809,956
Surgery	2,173	(24.9)	84,342	282,765	899,121	2,378	(18.9)	73,468	270,091	833,463
Anesthesia	2,308	(26.4)	4,034	21,685	128,490	1,302	(10.3)	3,206	37,804	137,275
Home medical care	1,674	(19.2)	92,845	170,719	255,956	2,516	(20.0)	92,047	160,600	230,400
Others	8,716	(99.7)	6,120	14,630	44,280	12,326	(97.9)	3,400	9,150	24,640
*B*. Clinical departments*										
Internal medicine	4,860	(55.6)	93,901	236,761	505,916	7,870	(62.5)	86,366	197,135	410,705
Orthopedic surgery	6,195	(70.9)	42,810	106,405	280,076	4,266	(33.9)	12,880	41,853	119,318
Ophthalmology	2,591	(29.6)	8,270	17,100	44,752	4,211	(33.4)	7,274	14,690	40,929
Dermatology	1,852	(21.2)	4,166	15,773	45,415	2,057	(16.3)	4,365	13,304	34,397
Otorhinolaryngology	928	(10.6)	6,700	13,794	36,741	2,198	(17.5)	10,096	24,961	69,487
Cardiology	1,100	(12.6)	41,235	160,150	373,968	1,652	(13.1)	53,528	170,749	423,445
Neurosurgery	1,035	(11.8)	21,460	67,363	234,508	1,332	(10.6)	20,973	43,667	176,078
Urology	1,108	(12.7)	12,458	42,868	96,491	1,240	(9.8)	12,407	34,358	83,718
Neurology	996	(11.4)	23,228	78,911	235,778	1,136	(9.0)	19,412	45,490	156,625
Diabetology	360	(4.1)	87,181	194,789	391,439	1,140	(9.1)	67,564	145,347	258,787
**Medication costs**										
Antidiabetic drugs										
*Glycemic control drugs*	4,185	(47.9)	16,811	56,503	92,070	7,185	(57.1)	18,950	56,695	87,990
*Any other antidiabetic drugs*	3,077	(35.2)	2,596	7,421	18,203	8,476	(67.3)	1,222	3,545	10,642
Analgesics										
*NSAIDs/acetaminophen*	7,254	(83.0)	2,030	8,330	22,636	6,995	(55.6)	408	2,317	10,395
*Drugs for neuropathic pain*	8,517	(97.4)	7,449	22,122	45,451	1,651	(13.1)	1,932	6,611	19,400
*Any other analgesics*	2,715	(31.1)	184	702	3,569	2,605	(20.7)	112	304	2,154
Anesthesia	4,348	(49.7)	108	316	2,939	4,342	(34.5)	92	207	1,022
Any other drugs	8,608	(98.5)	58,874	140,218	270,518	12,114	(96.2)	41,263	103,300	212,147

Each cost for the period of ± 6 months from the first diagnosis of pDPN or non-pDPN was summarized. Costs were expressed in Japanese yen (\).

Abbreviations: pDPN, painful diabetic peripheral neuropathy; non-pDPN, diabetic peripheral neuropathy without painful symptoms; NSAIDs, non-steroidal anti-inflammatory drugs.

*Clinical departments visited by >1,000 patients in either group were listed.

## Discussion

This was the first study to describe the characteristics of patients with pDPN and those with non-pDPN in Japan using a large-scale claims database. We found that patients with pDPN had more comorbidities and incurred higher medical costs than patients with non-pDPN. The differences in their characteristics may suggest that patients who develop pDPN may be clinically distinct from patients with non-pDPN.

To date, the comorbidity profiles of patients with DPN in relation to the presence or absence of painful symptoms have not been well documented. In this study, patients with pDPN had more orthopedic diseases, constipation, insomnia and gastric diseases than patients with non-pDPN. Identifying the causes of these comorbidities lay outside the scope of the study; however, some possible explanations can be provided. Type 2 DM lowers bone turnover [20], which may increase the risk for fracture or low back pain, although the underlying mechanisms have not yet been clarified, and constipation may be caused by diabetic autonomic neuropathy. Insomnia may be attributed to pDPN and painful musculoskeletal conditions such as low back pain and lumbar spinal stenosis [21–23], which were observed in >35% of patients with pDPN. Gastric ulcers and chronic gastritis may have developed as adverse effects of NSAIDs, which were prescribed to >60% of patients with pDPN. However, other mechanisms may have been responsible, and the relationships between these comorbidities and pDPN should be examined in more detail.

Although this study was unable to determine the causes of these comorbidities, the different comorbidity profiles suggest that patients with pDPN and patients with non-pDPN may be clinically distinct. Patients with pDPN may be subject not only to the complications of DM and painful symptoms, but also to other comorbid disorders, and the side effects of drugs given to treat pain and comorbid disorders. Despite such a high disease burden, pDPN tends to be under-diagnosed in clinical settings [8,9], possibly because patients tend not to complain about their pain. A study in the United Kingdom reported that 12.5% of patients with pDPN had not told their physician about their pain [24]. There may be a poor understanding of the relationship between pain and DM, as well as difficulty describing symptoms [8]. Physicians may need to be mindful of the characteristics of pDPN regardless of whether patients complain of pain, in order not to overlook this complication.

Glycemic control is important for the management of DPN [25,26]. Good glycemic control can prevent the development of DPN in type 1 DM [27], although recent reports have suggested that strict glycemic control may not be sufficient to prevent the development or progression of DPN in type 2 DM [28,29]. However, to date, the relationship between poor glycemic control and the development of pDPN has not been clarified. In this study, glycemic control status was similar between patients with pDPN and those with non-pDPN, and around 60% of patients in both groups exhibited satisfactory glycemic control (HbA1c <7%). The results not only support that other factors than hyperglycemia may play a role in the development of DPN in type 2 DM [30], but also suggest that hyperglycemia may be necessary but not sufficient to cause pDPN. The development of pDPN may not be prevented by glycemic control alone, thus other factors causing or exacerbating pDPN need to be identified in future studies.

We examined the medical costs for a period of one year for each group, and showed that the median total medical costs were higher in patients with pDPN than in patients with non-pDPN (\517,762 *versus* \359,909). Slightly a higher proportion of patients with pDPN required surgery and hospitalization compared with the non-pDPN group, implying that patients who develop pDPN may be more vulnerable to complications, for which medical costs would be incurred. A review of each patient’s claims data revealed that patients in the pDPN group tended to have more opportunistic infections and bladder problems. These results suggest that satisfactory glycemic control alone may not reduce the medical costs of patients with pDPN. Thus, it is important to establish the pathogenesis of pDPN and identify a therapeutic target. As microvascular complication is one of the factors to develop pDPN, patients may need a treatment targeting the improvement of blood circulation.

This study had several limitations. First, as data contained in the database were collected from acute-care hospitals using the DPC/PDPS, the study population may not represent the overall population of patients with DPN in Japan. In addition, glycemic control status was examined with a relatively small sample size, owing to the limited availability of laboratory data. Thus, the generalizability of our results may be limited. Second, as data were not linked to patients’ medical records, information such as severity and non-pharmacologic therapy (e.g. dietary therapy) could not be obtained. Because this study relied on claims data for disease name for pDPN or non-pDPN, the results of this study may not necessarily reflect the reality of treatment situations in actual patients with pDPN or non-pDPN. In addition, because pDPN tends to be under-diagnosed in clinical settings as discussed above, the possibility of under-reporting pDPN might also be inevitable in this study. Furthermore, as the database was hospital-based, treatment and medical histories recorded at different hospitals could not be obtained, which may have resulted in misclassification. Our results should therefore be interpreted with care. Third, as this was a cross-sectional study, causal relationships between each parameter and pDPN or non-pDPN could not be investigated. Fourth, as hyperglycemia is often asymptomatic—especially in the early stages of DM—relying on the date of diagnosis to calculate the duration of DM may not be accurate [31]. There may also have been discrepancies between the duration of DM obtained from the claims data and the actual duration, due to the characteristics of the database. For example, patients might already have been diagnosed as DM at different hospitals prior to the date of diagnosis recorded in this database. This may explain the relatively short duration of DM in our study cohort, compared with 10–11 years reported in other epidemiologic data on patients with DM in Japan [32,33]. However, as our cohort contained more patients with satisfactory glycemic control (approximately 60%) than other previously reported populations (ranging from 40–45%) [32,33], a proportion of patients in our study may indeed have had a short duration of DM. The influence of the duration and severity of DM on pDPN also requires further study.

## Conclusion

We found that patients with pDPN experienced greater clinical and economic burdens than patients with non-pDPN. Patients who develop pDPN may suffer not only from the complications of DM and pain, but also from other comorbid disorders and the side effects of analgesics and other drugs. Our findings will increase awareness among physicians of the need for more attention to this complication, which will facilitate detailed clinical examinations and appropriate management of patients with pDPN.

## Supporting information

S1 Table Classification and generic names of antidiabetic drugs. (DOCX)
