# Supplementary material for: Clinical and economic burdens experienced by patients with painful diabetic peripheral neuropathy: An observational study using a Japanese claims database
Source: PLoS One. 2017 Oct 27;12(10):e0187250. doi: 10.1371/journal.pone.0187250 (PMC5659791; doi:10.1371/journal.pone.0187250)
Supplement: S1 Table — (DOCX) [file pone.0187250.s001.docx]

**S1 Table. Classification and generic names of antidiabetic drugs.**

| **Category** | **Subcategory by drug class** | **Generic Name** |
| --- | --- | --- |
| Glycemic control drugs | Insulin | Insulin Aspart (Genetical Recombination) |
|  |  | Insulin Glargine (Genetical Recombination) |
|  |  | Insulin Glulisine (Genetical Recombination) |
|  |  | Insulin Degludec (Genetical Recombination) |
|  |  | Insulin Detemir (Genetical Recombination) |
|  |  | Insulin Lispro (Genetical Recombination) |
|  |  | Insulin Human (Genetical Recombination) |
|  | Glucagon-like peptide-1 (GLP-1) receptor agonists | Exenatide |
|  |  | Lixisenatide |
|  |  | Liraglutide (Genetical Recombination) |
|  | Sulfonylurea (SU) agents | Acetohexamide |
|  |  | Gliclazide |
|  |  | Glibenclamide |
|  |  | Glimepiride |
|  |  | Tolbutamide |
|  | Biguanides | Buformin Hydrochloride |
|  |  | Metformin Hydrochloride |
|  | Insulin sensitizers | Pioglitazone Hydrochloride |
|  | Alpha glucosidase inhibitors | Acarbose |
|  |  | Voglibose |
|  |  | Miglitol |
|  | Rapid-acting insulinotropic agents | Nateglinide |
|  |  | Mitiglinide Calcium Hydrate |
|  |  | Repaglinide |
|  | Dipeptidyl peptidase-4 (DPP-4) inhibitors | Anagliptin |
|  |  | Alogliptin Benzoate |
|  |  | Saxagliptin Hydrate |
|  |  | Sitagliptin Phosphate Hydrate |
|  |  | Teneligliptin Hydrobromide Hydrate |
|  |  | Vildagliptin |
|  |  | Linagliptin |
|  | Sodium-glucose co-transporter-2 (SGLT2) inhibitors | Ipragliflozin L‒Proline |
|  |  | Canagliflozin Hydrate |
|  |  | Dapagliflozin Propylene Glycolate Hydrate |
|  |  | Tofogliflozin Hydrate |
|  |  | Luseogliflozin Hydrate |
|  | Compounding agents | Alogliptin Benzoate, Pioglitazone Hydrochloride |
|  |  | Glimepiride, Pioglitazone Hydrochloride |
|  |  | Pioglitazone Hydrochloride, Metformin Hydrochloride |
|  |  | Voglibose, Mitiglinide Calcium Hydrate |
| Any other antidiabetic drugs | - | Epalrestat |
|  |  | Mecobalamin |
